# Age-related EBV positive clonal B-cell Lymphoid proliferation (EBV+-DLBCL)

**DOI:** 10.4317/jced.53314

**Published:** 2017-01-01

**Authors:** Eleni Georgakopoulou, Marina Doukas-Alexiou, Eleana Stoufi, Christos Kittas, Gerasimos Pangalis, George Laskaris

**Affiliations:** 1 DDS, MD, Msc, PhD, Molecular Carcinogenesis Group, Department of Histology and Embryology Medical School NKUA; 2 DDS, Msc, Athens Medical Center hospital, Marousi, Athens; 3 DDS, Msc, PhD, Euroclinic Hospital Athens & Oral Medicine at Harvard School of Dental Medicine; 4 Prof Histology, MD, PhD, Department of Histology and Embryology Medical School NKUA; 5 Prof Hematoloy MD, PhD, Athens Medical Center hospital, Psychiko Clinic; 6 Associate Prof Oral Medicine, MD, DDS, PhD, Medical School of Athens, NKUA

## Abstract

The Ebstein Barr virus(EBV), herpes virus 5 has been associated with lymphoproliferative disordrers. Age-related EBV+ B-LPD is defined as an EBV+ clonal B-cell lymphoid proliferation or EBV+-DLBCL developing in patients over the age of 40 years in the absence of any known immunodeficiency and without an underlying T-cell lymphoma1. We present a case of EBV+ clonal B-cell lymphoid proliferation.

** Key words:**Oral mucosa ulcer, EBV+-DLBCL, age related.

## Introduction

The Ebstein Barr virus(EBV), herpes Virus 5, is part of the family of herpes viruses- whose genome consists of a linear double-stranded DNA. It is a widespread virus and it is estimated that more than 90% of adults have come into contact with EBV (positive antibodies).

The virus is transmitted through oral secretions and following the first infection it remains latent in a small percentage of B lymphocytes for life. EBV has been associated with certain pathologies: 1. lymphomas in immunocompromised patients, 2. a significant number of solid tumors mainly of epithelial origin 3. lymphoproliferative diseases (LPD)of both B and T / NK cell origin in immunocompetent patients. Age-related EBV+ B-LPD is defined as an EBV+ clonal B-cell lymphoid proliferation or EBV+-(Diffuse Large B-cell Lymphoma) DLBCL developing in patients over the age of 40 years in the absence of any known immunodeficiency and without an underlying T-cell lymphoma (1). It was first described in 2007 in Japanese patients (2). It may develop in extranodal sites including the oral mucosa which is very rarely reported as a primary localization with only 12 cases having been reported in the literature (according to our knowledge) (3).

## Case report

**Figure 1:**
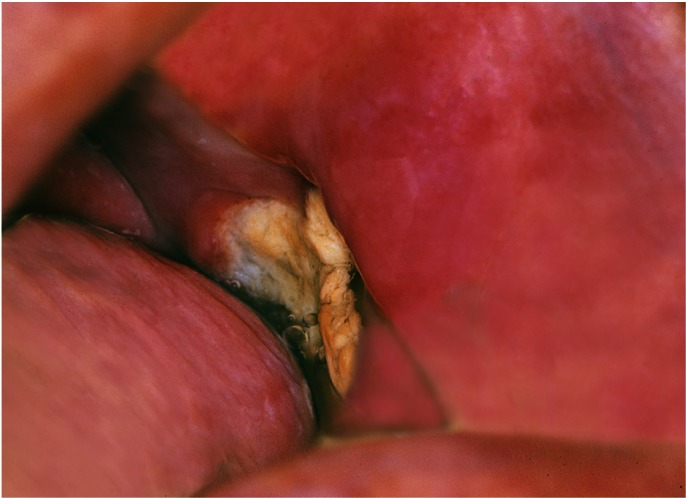
The oral ulcer.

This is a case of an 81 year old patient, with a history of coronary artery disease and diabetes mellitus, diagnosed with age related EBV-associated lymphoproliferative disorder. The localisation was extranodal in the form of a mucosal ulcer of 1×1 cm on the left buccal mucosa (Fig. 1).

The ulcer was first noticed during routine dental check up. The patient was referred to an Oral Medicine specialist for further evaluation (GL). The clinical differential diagnosis is shown on  Table 1. A biopsy was performed.

**Table 1:**
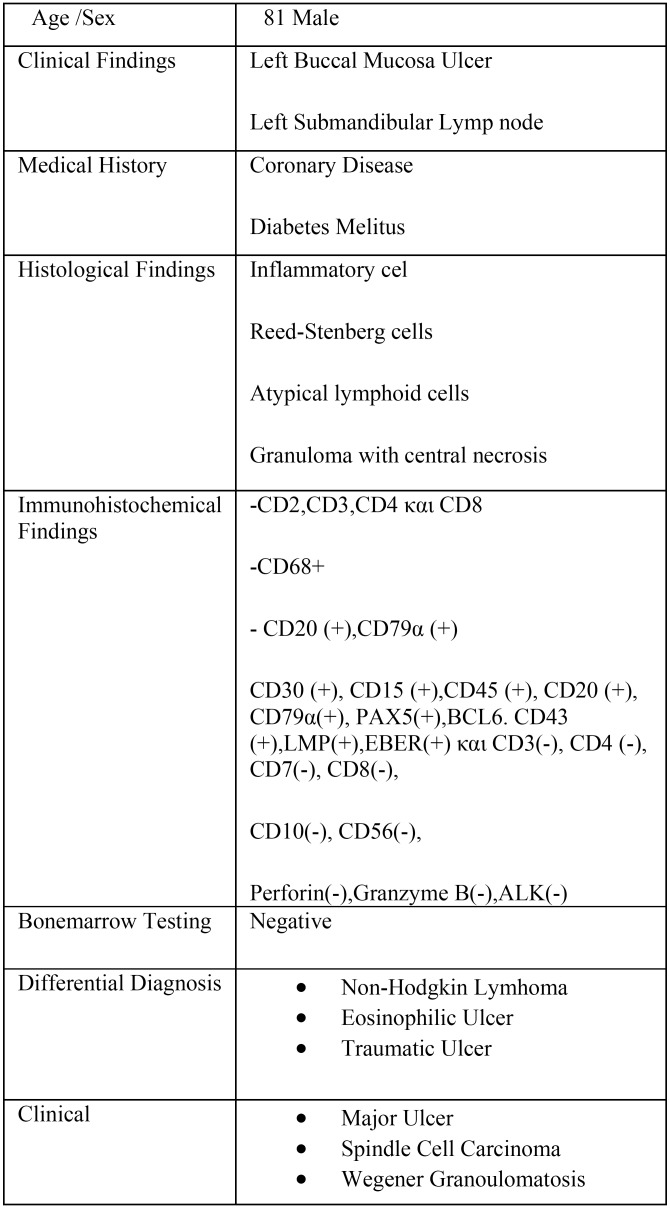
Case Description.

**Figure 2:**
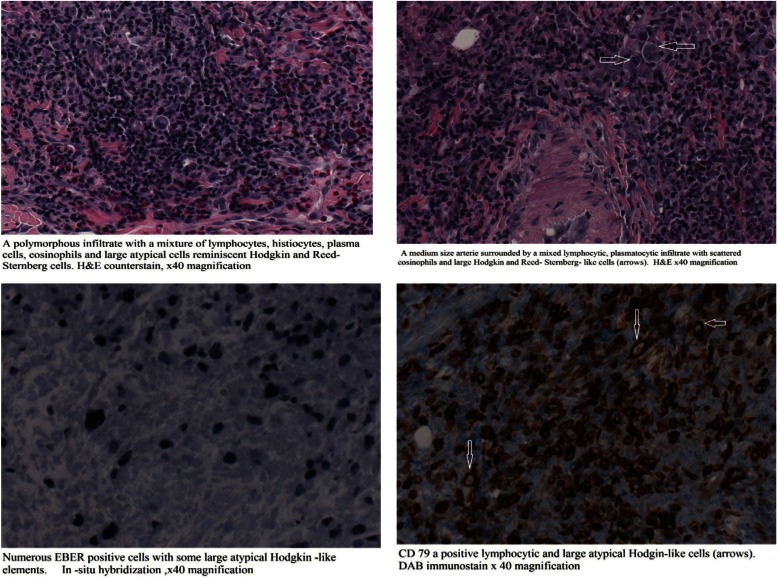
The histological and Immunohistochemical features of the ulcer.

Histologicaly an ulcer was observed with a diffuse infiltration of the lamina propria by consisting mixed cell population consisted of histiocytes, polymorphonuclear cells, many eosinophils, minor lymphocytes and mainly atypical major lymphoid cells. Additionally rare cells similar to Ηodgkin and Reed-Stenberg cells were located (Fig. 2). Angiocentric distribution of atypical lymphoid elements was observed. Also a sizeable granuloma with central necrosis in which there were clusters of polymorphonuclear and histiocytes with peripheral epithilioid cells was recognised. The histochemical examination was negative for bacteria or mycobacteria, fungi, and other microorganisms. (Dyes: Ziehl-Nielsen, GMS, PAS KAI Giemsa).

The histological and immunohistochemical examination suggested that the ulcer was probably age related, EBV-associated lymphoproliferative disorder.

The patient was referred to a haematology clinic for further evaluation.

Upon arrival, the patient was asymptomatic and physical examination revealed a solitary submandibular lymph node on the left side with maximum diameter of 2cm, and local swelling of the left side of the face (~ 2cm). These findings were confirmed by Computed Tomography (CT) chest and upper and lower abdomen CT.

Laboratory and haematological tests showed: Anemia (Hct: 36.5%) normochromic, normocytic , elevated ESR (63mm) and CRP (1.33mg / dl vn <0.7). Aspiration of the bone marrow including : immunophenotyping formulation of bone marrow cells as well as bone marrow histological examination and immunohistochemistry showed no infiltration from lymphoma. (clinical and laboratory findings are summarized in  Table 1.

## Discussion

The age related, EBV-associated lymphoproliferative disorder entity is not included in the Classification of Neoplastic Diseases of the Hematopoietic and Lymphoid Tissues World Health Organization (WHO) in 2008. Histology shows complete absence of normal tissue and nodal structure with the domination of large atypical lymphoid cells/immunoblasts and Hodgkin/Reed-Sternberg-like giant cells with variable amounts of inflammatory cells in the surroundings 4The proportion of neoplastic to inflammatory cells, the quantity of mitotic cells and the level of necrosis may vary significantly. As a result EBV+ DLBCL of the elderly was classified into low grade polymorphic and high grade monomorphic lymphoma types (4). Further studies have shown both types to be different ends in the spectrum of disease, and are all high grade lymphomas (4). The neoplastic large lymphoid cells show expression of CD20/CD79a and PAX-5, with variable expression of CD30, LMP-1 and EBNA-2. On the other hand CD15, CD10 and BCL6 are generally negative. Neoplastic cells show EBER positivity and high Ki-67 expression.

Differential diagnosis includes EBV+ B-LPD classical Hodgkin lymphoma and EBV–DLBCL and EBV+ DLBCL of the elderly which are highly aggressive with a median survival of 2 years. These patients are less responsive to standard chemotherapy com-pared with other B-LPD. The type of mucocutaneous ulcer has been described by Dojcinov SD *et al.* (5) as a separate entity of the newly introduced subgroup of lymphoproliferative diseases by name: Epstein-Barr virus (EBV) positive diffuse large B cell lymphoma (DLBCL) of the elderly. It has been suggested that the disorder may be due to a minimum and limited to a small area failure in immunosurveillance over EBV5. This subtype has a better prognosis. The review of the literature is limited with rare references concerning this subgroup of lymphomas with localization in the oral cavity (2). It would be interesting to evaluate further the genetic and epigenetic changes in these specific cases of localised oral lymphomas as they could constitute a completely separate category. In addition as the general population shows an improved median age of death it is expected that such cases of age related pathologies will be more common and clinicians should be aware in order to offer their patients the best care possible.

The Oral Medicine Specialist must have a broad medical background and experience so as to diagnose atypical clinical lesions.
